# Investigating the Impact of the Bit Depth of Fluorescence-Stained Images on the Performance of Deep Learning-Based Nuclei Instance Segmentation

**DOI:** 10.3390/diagnostics11060967

**Published:** 2021-05-27

**Authors:** Amirreza Mahbod, Gerald Schaefer, Christine Löw, Georg Dorffner, Rupert Ecker, Isabella Ellinger

**Affiliations:** 1 Institute for Pathophysiology and Allergy Research, Medical University of Vienna, A-1090 Vienna, Austria; christineloew1203@outlook.com (C.L.); isabella.ellinger@meduniwien.ac.at (I.E.); 2 Department of Computer Science, Loughborough University, Loughborough LE11 3TT, UK; gerald.schaefer@ieee.org; 3 Section for Artificial Intelligence and Decision Support, Medical University of Vienna, 1090 Vienna, Austria; georg.dorffner@meduniwien.ac.at; 4 Department of Research and Development, TissueGnostics GmbH, 1020 Vienna, Austria; rupert.ecker@tissuegnostics.com

**Keywords:** bit depth, nuclei segmentation, fluorescence staining, medical image analysis, computational pathology, deep learning

## Abstract

Nuclei instance segmentation can be considered as a key point in the computer-mediated analysis of histological fluorescence-stained (FS) images. Many computer-assisted approaches have been proposed for this task, and among them, supervised deep learning (DL) methods deliver the best performances. An important criterion that can affect the DL-based nuclei instance segmentation performance of FS images is the utilised image bit depth, but to our knowledge, no study has been conducted so far to investigate this impact. In this work, we released a fully annotated FS histological image dataset of nuclei at different image magnifications and from five different mouse organs. Moreover, by different pre-processing techniques and using one of the state-of-the-art DL-based methods, we investigated the impact of image bit depth (i.e., eight bits vs. sixteen bits) on the nuclei instance segmentation performance. The results obtained from our dataset and another publicly available dataset showed very competitive nuclei instance segmentation performances for the models trained with 8 bit and 16 bit images. This suggested that processing 8 bit images is sufficient for nuclei instance segmentation of FS images in most cases. The dataset including the raw image patches, as well as the corresponding segmentation masks is publicly available in the published GitHub repository.

## 1. Introduction

The morphology of nuclei regulates and, vice versa, is regulated by the environment, as well as the activity of the cell. Nuclei parameters are important measures for cell biologists investigating physiological and pathophysiological processes and are used clinically for, e.g., the diagnosis of malignant and other diseases [1]. Physiologically, nuclear morphology can for instance be dramatically influenced during the differentiation of immune cells [1]. In cytopathology, as well as surgical pathology, assessing the morphologic abnormalities of nuclei is essential for the diagnosis of malignancies. Malignant transformation of cells can result in larger nuclei and an increased nuclear-to-cytoplasmic ratio. The chromatin of malignant cells can be altered, and nuclear membrane irregularities such as thickening, dents, folds, grooves and pseudo inclusions can be observed. Malignant cells may display a combination, but not necessarily all of these morphologic abnormalities [2]. Viral infections of cells can also affect the morphology of the nucleus of the host cell. Among the most common morphological alterations of the nucleus observed due to viral infection of cells are the disruption of the nuclear membrane and fragmentation of the nucleus. Many viruses are also involved in re-localization or depletion of host nucleolar proteins or alterations of certain nuclei substructures, such as the promyelocytic leukaemia nuclear bodies [3]. Visualisation of nuclei in tissue and cell samples can be performed via either chemical dyes including hematoxylin or via fluorescent dyes such as 4${}^{\prime }$,6-diamidin-2-phenylindole (DAPI) [1,4]. Fluorescence imaging is widely used in cell biology and biomedical research, and the identification of the cell nucleus is an important first step in the quantitative analysis of fluorescence images [5].

The development of automated computer-based approaches for nuclei instance segmentation as a prerequisite for subsequent image analysis has been an active research area in recent years [6]. Standard image-processing techniques such as Otsu thresholding or watershed-based algorithms [7,8] or classical machine learning approaches such as random forest classifiers are among the proposed approaches for this task [9,10]. Although these methods are rather fast at performing segmentation and can be easily implemented by non-computer scientists through publicly available software such as ImageJ [11] or CellProfiler [12], their performances degrade drastically in challenging cases [13]. Up to this point, supervised deep learning (DL)-based methods and, more specifically, convolutional neural networks (CNNs) have delivered the best nuclei instance segmentation performances in microscopic images [6,10,13].

In general, three types of CNN-based algorithms were proposed in previous studies to perform nuclei instance segmentation, namely distance-based algorithms, which try to find the Euclidean distance maps of each individual object in the images, ternary segmentation-based algorithms, which assign an extra class for the nuclei boundaries, and localization-based algorithms, which try to find the nuclei location and then perform segmentation [6]. In some more recent studies, combinations of these methods were employed to further enhance the nuclei instance segmentation performance [14,15,16,17]. However, most of these algorithms were developed and tested on images where the nuclei were counterstained with chemical dyes such as hematoxylin, and only a few studies have analysed nuclei instance segmentation in images with fluorescence-stained (FS) nuclei [10,18,19]. One main issue that has hampered the development of supervised DL approaches for nuclei instance segmentation of FS images is the limited number of publicly available fully annotated FS image datasets in comparison to the image datasets with hematoxylin and eosin (H&E) or immunohistochemically (IHC) stained samples, where nuclei are chemically labelled. Parts of the Kaggle Data Science Bowl 2018 dataset [20], Kromp et al. dataset [5], NucleusSeg dataset [21] and Caicedo et al. dataset [10,22] are among the few publicly available FS image databases that can be used for developing computer-based nuclei instance segmentation algorithms.

For H&E-stained and IHC-stained images, the most conventional image format is the 24 bit RGB images (eight bits per colour channel). For fluorescent applications, more and more cameras are available that can acquire even 16 bit images. There are general recommendations that more bits give more information and precision in the images [23,24]. The acquisition of 16 bit images is sometimes even recommended when the images are later rescaled to 8 bit images for image processing [23]. In particular, in cases where there is low contrast between the stained objects and the background, sixteen-bit image capturing enables the identification of subtle changes in the images.

Other sources, in contrast, suggest that the lower fluorescence signal levels are better suited to 8 or 12 bit images rather than 16 bit images [24,25]. The acquisition of 16 bit images may have some additional drawbacks. In digital pathology labs where several hundreds of slides can be scanned per day, storing digitised images at a 16 bit image depth needs at least twice as much physical storage space in comparison to 8 bit images [25,26]. Moreover, a higher bit depth can reduce the imaging speed of a particular camera as more possible grey levels exist to convert the signals [24]. Due to the biological limitations of eyes, monochrome images are typically viewed at 8 bit, even if the image was acquired at a higher bit depth [24].

With respect to image analysis, it is unknown whether 16 bit images allow for a significant better nuclei instance segmentation performance in comparison to 8 bit images. To our knowledge, no study has been conducted to investigate this impact, and most of the (immuno) FS image datasets released so far can not be used for such an investigation as they contain only 8 bit images. The main contributions of this work are twofold. Firstly, we released a fully annotated nuclei instance segmentation dataset of 16 bit FS images at different image magnifications and from five different mouse organs to support the development of computer-based nuclei instance segmentation algorithms. Secondly, we investigated the impact of image bit depth (i.e., eight bits vs. sixteen bits) on the nuclei instance segmentation performance using our proposed dataset and a second publicly available dataset.

## 2. Materials and Methods

### 2.1. Datasets

Our released dataset (referred to as the BitDepth dataset in this paper) contains fully annotated FS images for nuclei instance segmentation. Heart, kidney, liver, muscle (M. soleus) and bone (femur) were obtained from 8 week-old male C57BL/6J mice [27]. Tissues were formaldehyde-fixed and paraffin-embedded. Four-micrometre sections were stained with DAPI and cover-slipped using Fluoromount-G mounting medium. Whole-slide images (WSIs) were generated with a TissueFAXS (TissueGnostics, Vienna, Austria) scanning system composed of an Axio Imager Z1 (Zeiss, Oberkochen, Germany), equipped with the Plan-Neofluar 20×/0.5 objective (referred to as 20×), the Plan-Neofluar 40×/0.75 objective (referred to as 40× air), the 40×/1.3 oil immersion objective (referred to as 40× oil), as well as the Plan-Apochromat 63×/1.4 oil immersion objective (referred to as 63× oil) and a 49000 ET-DAPI filter set (Chroma Technology Corp, Vermont, USA) in combination with the TissueFAXS Image Acquisition and Management Software (Version 6.0). Using a monochrome camera (Hamamatsu, Hamamatsu, Japan), grayscale images were acquired at 16 bit resolution using the four different objectives (20× air, 40× air, 40× oil and 63× oil with pixel sizes of 0.32 m, 0.16 m, 0.16 m and 0.10 m, respectively). A senior cell biologist selected candidate fields of view (FOVs) with a fixed size of 2048 × 2048 pixels to perform manual nuclei instance segmentation to form the dataset. Central image cropping was then applied on the selected FOVs with a fixed size of 512 × 512 pixels.

**Figure 1:**
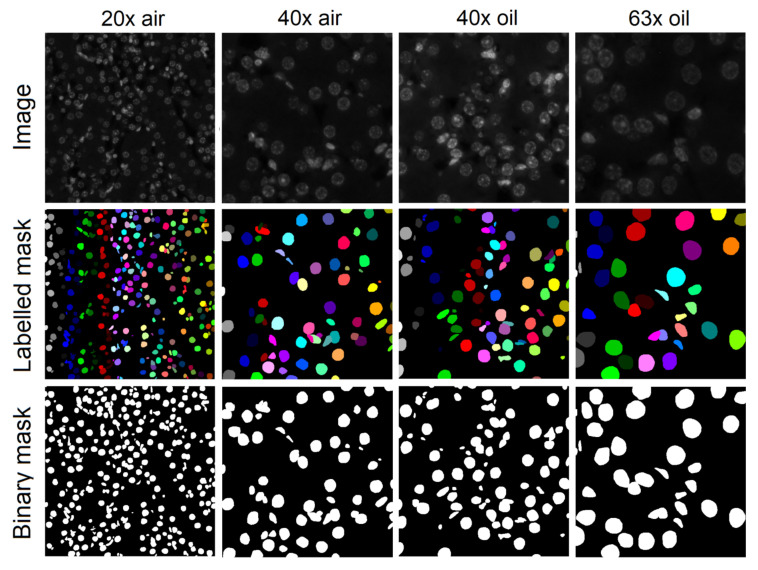
Examples of kidney images at different image magnifications with their corresponding labelled and binary segmentation masks.

Manual instance segmentation of the BitDepth dataset was performed as described recently in [28]. Example kidney image patches acquired with different objectives with their corresponding labelled and binary masks are depicted in Figure 1. Besides the conventionally labelled and binary masks, we also generated other auxiliary segmentation masks that can be utilised in the development of CNN-based instance segmentation models. The auxiliary segmentation masks included Euclidean distance transforms, weighted maps that give higher weights to the touching borders and border-removed binary segmentation masks. More details of the dataset are listed in Table 1. Our fully annotated dataset, as well as related codes to generate segmentation masks from the ImageJ ROI files are publicly available in the related GitHub repository (https://github.com/masih4/BitDepth_NucSeg (accessed on 26 May 2021)).

To confirm the generalisation of the results, besides our proposed dataset, we used another publicly available dataset presented in [10]. The Caicedo et al. dataset contains 200 FS images of U2OS cells acquired with a 20× objective. More than 23,000 nuclei were manually segmented in this dataset with an average of 118.1 nuclei per image. The dataset includes image patches extracted from WSIs with a fixed size of 520 × 696 pixels at 16 bits. Further details about this dataset can be found in [10,22].

While the Caicedo et al. dataset has more manually segmented nuclei compared to the BitDepth dataset, it only contains images with one image magnification and from one cell type, which is not optimal to be used alone, as it may not deliver generalisable results. In contrast, our dataset contains images with three image magnifications (20×, 40× and 63×) and from five mouse organs. To our knowledge, other publicly available datasets of FS images for nuclei instance segmentation only include images at 8 bits and thus cannot be used in this study to investigate the impact of image bit depth on the nuclei instance segmentation performance.

### 2.2. Pre-Processing

For the BitDepth dataset, we created four sub-datasets (Dataset 1, Dataset 2, Dataset 3 and Dataset 4) from the acquired raw 16 bit images for each image magnification. In Dataset 1, we divided all intensity values in the images by 65,535. In Dataset 2, we applied outlier removal on the normalised derived images. The reason that we applied the outlier removal pre-processing step was the existence of saturated pixels in the images (very bright spots) due to acquisition artefacts. To remove outliers, we calculated the 99th-percentile intensity value for each image as the threshold. Then, all intensity values above the calculated threshold were cut off, and their intensity values were replaced by the derived threshold. The utilised percentile value was selected empirically to not change the raw images drastically and at the same time remove the outliers from the images. To create Dataset 3, we used the following formula to normalise the images:

(1) $$X_{norm}=\frac{X_{raw}-X_{min}}{X_{max}-X_{min}}$$

where $X_{raw}$ represents the raw intensity values in a 16 bit image and $X_{min}$ and $X_{max}$ are the minimum and maximum intensity values in the 16 bit image, respectively. To create Dataset 4, we used the Dataset 3 images and applied the outlier removal step described above.

To create raw 8 bit images, we used the following formula and applied it to raw 16 bit images.

(2) $$X_{8bit}=uint8\left[\frac{X_{16bit}}{256}\right]$$

where uint8 is the round function to assign all values to integer numbers between 0 and 255 to form raw 8 bit images from 16 bit images. From these raw 8 bit images, we also created Datasets 1–4.

**Figure 2:**
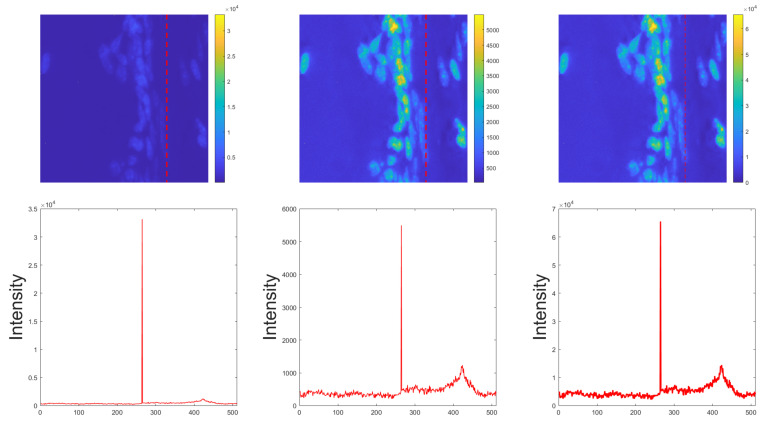
Example of raw, outlier-removed and normalised 16 bit images of a bone sample at 40× magnification from left to right in the first row, respectively. We also plot a profile intensity line (depicted with dashed red lines in the images) in the second row. The raw 16 bit image was selected from the BitDepth dataset.

**Figure 3:**
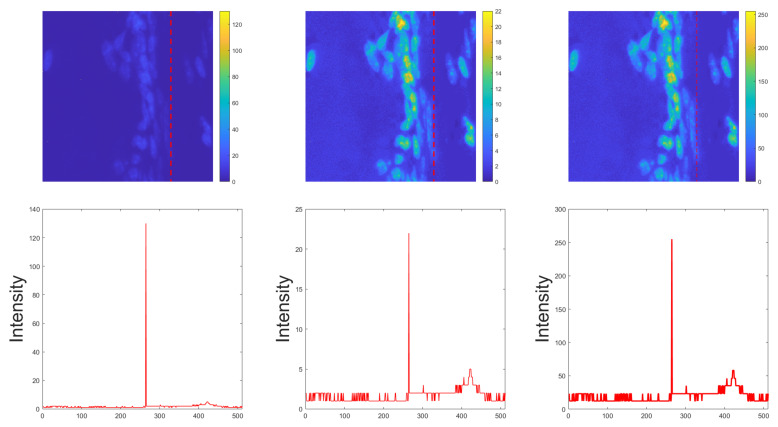
Example of raw, outlier-removed and normalised 8 bit images of a bone sample at 40× magnification from left to right in the first row, respectively. We also plot a profile intensity line (depicted with dashed red lines in the images) in the second row. The raw 8 bit image was selected from the BitDepth dataset and corresponds to the 16 bit image presented in Figure 2.

Examples of raw, outlier-removed and normalised 16 bit and 8 bit images of a bone sample at 40× magnification are depicted in Figure 2 and Figure 3, respectively.

For the Caicedo et al. dataset, we created a normalised 16 bit dataset and a normalised 8 bit dataset similar to what we did for Dataset 4 in the BitDepth dataset. We used these two derived datasets to perform additional experiments to explore the generalisability of the obtained results from the BitDepth dataset.

Finally, we created other sub-datasets of 2, 4, 6, 10, 12 and 14 bit images from both the BitDepth and the Caicedo et al. datasets. To create these sub-datasets, we used min/max normalisation with outlier removal with the identical setting as Dataset 4. Although using these bit depths is not very common in practice in comparison to 8 bit and 16 bit images, it allowed us to investigate the impact of other image bit depths on the nuclei instance segmentation performance.

### 2.3. Nuclei Segmentation Method

To investigate the impact of the image bit depth of FS images on the performance of nuclei instance segmentation, we employed our recently published algorithm for nuclei segmentation in H&E-stained microscopic images [16]. This algorithm consisted of two training stages for background detection and distinguishing overlapping nuclei. For background detection in the first stage, a U-Net-like encoder–decoder model was trained. Binary segmentation masks were used in this stage. In the second stage, a regression U-Net model was trained to predict Euclidean distance transforms of all individual objects in the images. Euclidean distance transforms derived from the labelled masks were used in the second stage of training. The results from these two stages were then merged through a watershed-based method [8] to form nuclei instance segmentation masks. As the original architecture of the utilised approach was designed for H&E-stained RGB images, we copied the single-channel FS microscopic images three times to have three channels as the input of the network. Further details about the model and merging procedure can be found in [16]. We used the Adam optimiser, a learning scheduler (starting learning rate of 0.01, which halved after every 8 epochs) and a batch size of 4 to train the models. The models in each stage were trained for 30 epochs. To partially overcome the issue of limited training images in the BitDepth and Caicedo et al. datasets, we utilised intensity-based and morphological data augmentation techniques including random brightness and contrast shift, random horizontal and vertical flipping and random rotations, as suggested in [29,30]. The utilised augmentation techniques were only applied on the training images and not on the validation set images. The utilised model had around 7.7 million trainable parameters for each stage, which yielded an approximate total number of 15.2 million trainable parameters for both stages.

### 2.4. Evaluation

For the evaluation of the nuclei instance segmentation performance, we used three evaluation indexes, namely the similarity Dice score (Equation (3)), the aggregate Jaccard index (AJI) (Equation (4)) and the panoptic quality (PQ) score (Equation (5)) [6,31,32]:

(3) $$Dice=\frac{2.S\cap G}{S+G}$$

(4) $$AJI=\frac{\sum_{i=1}^{L}G_{i}\cap {\tilde{S}}_{K}\left(i\right)}{\sum_{i=1}^{L}G_{i}\cup {\tilde{S}}_{K}\left(i\right)+\sum_{l\in U}S_{l}}$$

(5) $$PQ=\frac{2.TP}{2.TP+FN+FP}\times \frac{\sum_{(G_{i},S_{i})\in TP}Jaccard\left(G_{i},S_{i}\right)}{TP}$$

where in the above formulas for a given image, *S* represents the predicted segmentation mask, *G* represents the ground truth, *L* is the total number of objects in *G*, *U* is the indices for the nuclei in *S* that have not been matched with any nuclei in *G*, ${\tilde{S}}_{K}\left(i\right)$ is the detected nucleus in *S* that maximises the Jaccard index with the $G_{i}$ in the ground truth, TPs are the matched pairs between *S* and *G*, FNs are the unmatched *G* instances and FPs are the unmatched *S* instances. In general, the Dice score indicated the semantic segmentation performance (i.e., separating foreground and background), while the AJI and PQ scores showed the instance segmentation performance (i.e., the ability of the model to separate touching objects).

To perform statistical tests, we used the Wilcoxon signed-rank test method for each of the evaluation indices [33,34]. In our initial experiments, we considered 0.05 as the threshold for the significance level. However, as we performed three statistical tests for each of the paired datasets, the level of significance could be reduced to 0.017 according to the Bonferroni correction [35].

### 2.5. Implementation

The manual instance segmentation was performed with ImageJ software (Version 1.52a) and its pre-built region of interest (ROI) tool. To convert ImageJ ROI files to conventional binary and labelled masks and also to create auxiliary segmentation masks, we employed MATLAB (Version 2020a). To develop the nuclei instance segmentation model, we used the Keras deep learning framework (Version 2.3.1) with a TensorFlow (Version 1.14) backend. All experiments were conducted on a single workstation with 32 GB of RAM, an Intel Core i7-8700 3.20 GHz CPU and a TITAN V NVIDIA GPU card.

### 2.6. Experimental Setup

To compare the nuclei instance segmentation performance of the models trained with 16 bit FS images and 8 bit FS images for the BitDepth dataset, images obtained with different objectives (20×, 40× air, 40× oil and 63× oil) were analysed separately. For each magnification, we performed eight experiments based on the image bit depth (i.e., 8 bits or 16 bits) and the utilised sub-dataset (i.e., Dataset 1, Dataset 2, Dataset 3 and Dataset 4, as explained in Section 2.2). Thus, we performed 32 experiments with identical settings, as explained in Section 2.3. To report the results for each experiment, we performed five-fold cross-validation with a fixed randomised seed point to have a fair comparison of the results.

In the next step, we performed two additional experiments based on the entire BitDepth dataset and using the min/max normalisation with the outlier removal step. For those two experiments and to merge the datasets, we resized the images of 20× and 40× magnifications to bring them to a similar resolution space (63×) and then performed five-fold cross-validation. The 20× images were resized to 1612 × 1612 pixels, and the 40× images were resized to 806 × 806 pixels. We performed random image cropping with a fixed size of 512 × 512 pixels to train the models for these two experiments. In the inference phase, 1612 × 1612 and 806 × 806 pixel images were resized to 1600 × 1600 and 800 × 800 pixel images, respectively, to be able to send them to the trained models.

Moreover, to investigate the impact of image bit depth on an external dataset, we used the two derived datasets from the Caicedo et al. dataset as explained in Section 2.2. We performed five-fold cross-validation for each of the derived datasets to measure the instance segmentation performance for 8 bit and 16 bit images.

Finally, in our last set of experiments for both the BitDepth and Caicedo et al. datasets, we used the created sub-datasets for other image bit depths (i.e., 2, 4, 6, 10, 12 and 14 as explained in Section 2.2) to investigate the impact of other depths on the nuclei instance segmentation performances.

## 3. Results

The derived results for the comparison of the nuclei instance segmentation performance of 8 bit and 16 bit images for the BitDepth dataset are shown in Table 2. In cases where statistical tests between 8 bit and 16 bit images led to *p*-values less than 0.05, the results are shown in bold in the table. The exact *p*-values for the experiments are shown separately in Table 3. It is worth mentioning that besides these experiments, we also performed additional examinations without any pre-possessing to train the segmentation model. However, we noted that for some folds of cross-validation, the training did not converge, and as a consequence, we did not report the results for those experiments.

**Figure 4:**
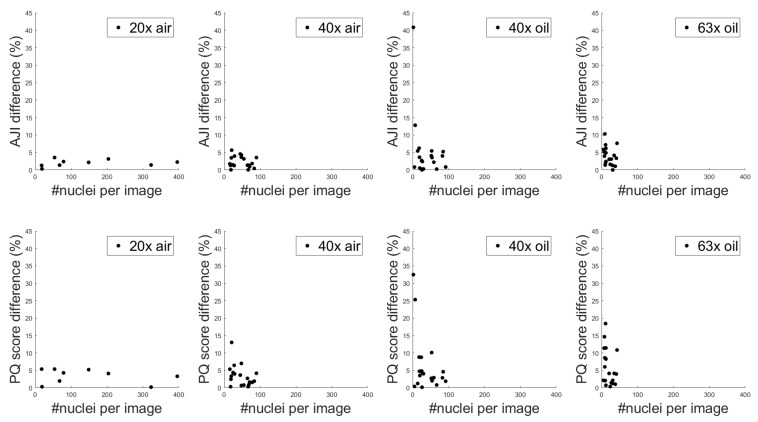
Differences between the AJI and PQ scores (%) for 8 bit and 16 bit images based on the number of nuclei in the images for different image magnifications in the BitDepth dataset. For each magnification, Dataset 4 was used to measure the performance differences between 8 bit and 16 bit images.

To have a more in-depth analysis of the segmentation performance differences between 8 bit and 16 bit images, we also plotted the absolute differences in instance-based scores against the number of nuclei inside the images for different image magnifications in Figure 4.

**Figure 5:**
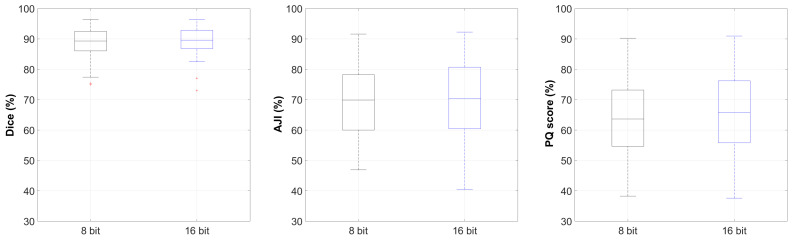
Nuclei instance segmentation performance boxplots for Dice, AJI and PQ scores derived from the BitDepth dataset (Y-axes are limited for a better visualisation, and hence, some outliers are not shown).

The results from the experiments that utilised the entire BitDepth dataset showed very competitive segmentation performances for both 8 bit and 16 bit images. An average Dice score of 88.7 ± 4.6%, an average AJI of 69.3 ± 12.3% and an average PQ score of 63.2 ± 12.7% were obtained for 8 bit images, while an average Dice score of 89.2 ± 4.4%, an average AJI of 70.6 ± 12.1% and an average PQ score of 64.9 ± 13.5% were obtained for 16 bit images. Boxplots of these experiments for different evaluation indexes are shown in Figure 5.

**Figure 6:**
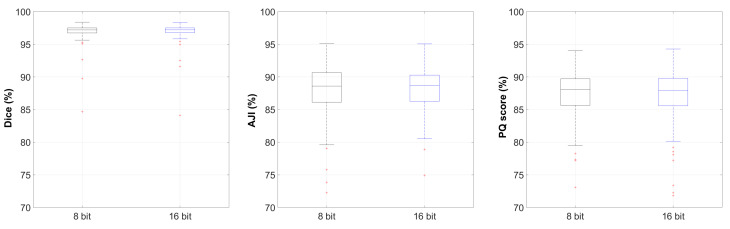
Nuclei instance segmentation performance boxplots for Dice, AJI and PQ scores derived from the Caicedo et al. dataset [10] (Y-axes are limited for better visualisation, and hence, some outliers are not shown).

**Figure 7:**
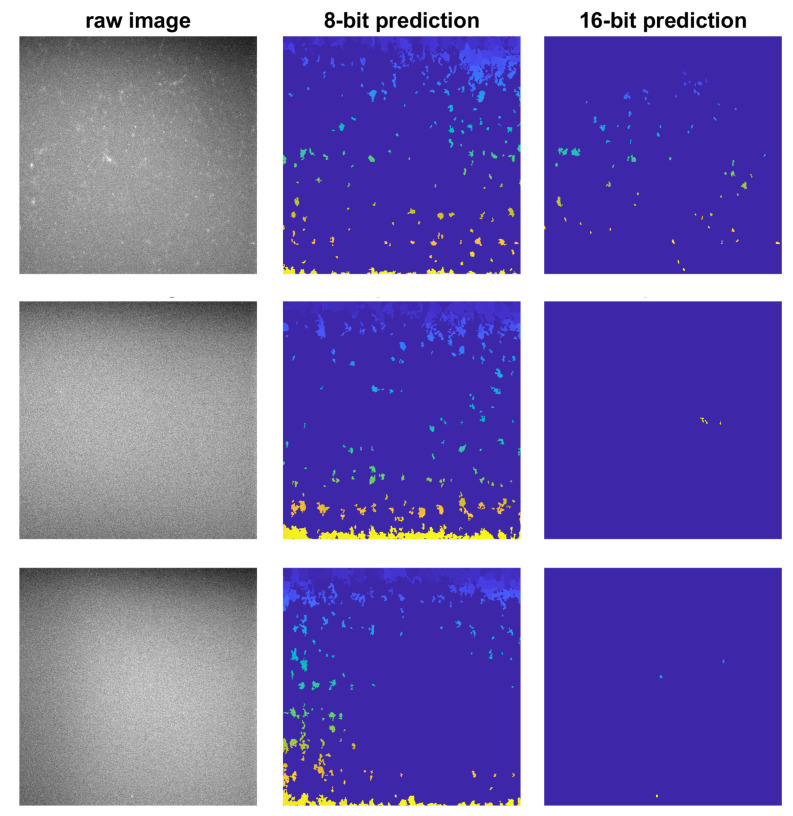
Three images from the Caicedo et al. dataset [10] with zero Dice, AJI and PQ scores. There were no nuclei in the ground truth segmentation masks for these three cases.

Results obtained from the Caicedo et al. dataset also showed very competitive segmentation performances with an average Dice score of 95.5 ± 11.9%, an average AJI of 86.0 ± 13.0% and an average PQ score of 85.6 ± 12.8% for 8 bit images in comparison to an average Dice score of 95.6 ± 11.9%, an average AJI of 86.0 ± 12.9% and an average PQ score of 85.7 ± 12.5% for 16 bit images. Boxplots of these experiments for different evaluation indices are shown in Figure 6. We note that for three cases in the Caicedo et al. dataset, zero Dice, AJI and PQ scores were achieved. For those cases, there were no nuclei in the ground truth segmentation masks, while the model predicted at least one nucleus, and according to Equations (3)–(5), zero scores were derived. Those three images, as well as their prediction segmentation masks are shown in Figure 7. If we disregarded these three images and recalculated the evaluation indexes, an average Dice score of 97.0 ± 1.2%, an average AJI of 87.3 ± 7.6% and an average PQ score of 86.8 ± 7.2% were obtained for 8 bit images, while for 16 bit images, an average Dice score of 97.0 ± 1.2%, an average AJI of 87.3 ± 7.3% and an average PQ score of 86.9 ± 6.6% can be obtained.

**Figure 8:**
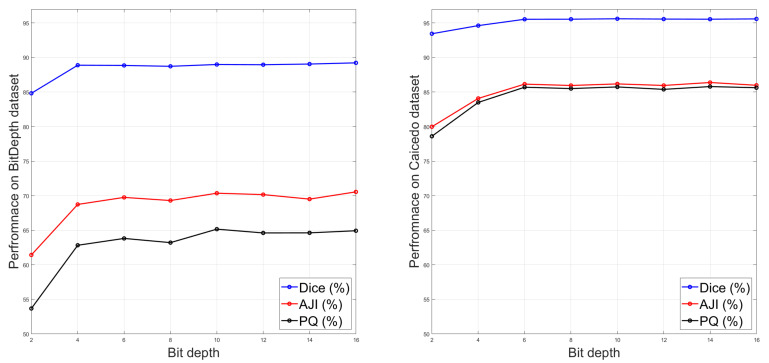
The average nuclei instance segmentation performance based on Dice, AJI and PQ scores for different image bit depths for the BitDepth and Caicedo et al. datasets.

The nuclei segmentation results from the experiments for additional image bit depths (2, 4, 6, 10, 12 and 14) for the BitDepth and Caicedo et al. datasets are shown in Figure 8. We also included the average results from the 8 bit images and 16 bit images as baselines to compare the segmentation performances in the figure.

## 4. Discussion

The main findings of this study are displayed in Table 2. As shown in the table, for each of the datasets and each image magnification, we observed very competitive nuclei instance segmentation performances for 8 bit and 16 bit images. More specifically, for the average Dice scores, the results were very close with less than 1% performance differences for the 8 bit- and 16 bit-derived datasets. Although in some cases, the average results were rather different for the AJI and PQ scores, in most cases, the differences were not significant. The differences in PQ and AJI were more striking at higher image magnifications. As reported in Table 2 and Table 3 and considering a threshold level of 0.05, in six out of thirty-two pair results for the Dice, AJI and PQ scores, the results were statistically different. If we used Bonferroni correction and reduced the level of significance to 0.017, only in two out of thirty-two tests, the results were different. However, Bonferroni correction especially in our case where statistical tests for the Dice, AJI and PQ scores were correlated may not be applicable [36,37]. The competitive segmentation performance of 8 bit and 16 bit images suggested that the model relied on the nuclei morphological features rather than exact intensity values of background and foreground pixels to segment the images, and hence, the model was not sensitive to subtle changes in intensity values and delivered comparable segmentation performance for 8 bit and 16 bit images. Besides the average scores and standard deviations (SDs) are also reported for all experiments in Table 2.

As mentioned in the Results Section, training segmentation models without any pre-processing step did not lead to training convergence, which showed the importance of the normalisation step in training a DL-based algorithm. By comparing the results derived from different pre-processing steps (Dataset 1 to Dataset 4), competitive segmentation performances can be observed. However, the average results derived by the min/max normalisation with outlier removal (Dataset 4) were slightly superior compared to other normalisation steps for both 8 bit and 16 bit images.

As the results for the sub-datasets in Table 2 and also the results for the entire BitDepth dataset in Figure 5 showed, the SDs were rather large for the AJI and PQ scores. However, the large SDs were related to the level of the image complexity for segmentation. In other words, some images were easier to segment, which led to high segmentation scores, and some images were more challenging to segment, thus resulting in low segmentation scores. However, for the Dice score, the algorithm can deliver rather good semantic segmentation performance across all images. This led to the low level of SDs for the Dice scores for the BitDepth dataset.

To further investigate the differences in the segmentation performances for different image magnifications, we performed additional analysis to relate the number of nuclei in an image to the difference in the nuclei instance segmentation performance, as shown Figure 4. In our released BitDepth dataset, we had many more nuclei per image for lower image magnifications (e.g., 145.5 for 20× air) in comparison to higher image magnification (e.g., 19.4 for 63× oil). In the case of the existence of only a few nuclei inside an image, the incorrect split/merge of nuclei, even for one or two instances, can change the instance-based results greatly. To measure this impact quantitatively, we plot the differences in instance-based scores against the number of nuclei inside the images for different image magnifications in Figure 4. As the results in Figure 4 imply, the differences of the results were much more evident when only a few nuclei existed in the images (for instance by comparing the 20× air magnification results where the differences were below 6% with the 63× oil magnification results where the differences for some cases were above 10%). However, this issue was not related to image bit depth or FS images and can appear in other staining types where the number of nuclei per image is very small.

To confirm the generalisability of our interpretations, we performed additional experiments on the Caicedo et al. dataset. As the results in Figure 6 show, the nuclei instance segmentation performances for this dataset were also very competitive for 8 bit and 16 bit images, in agreement with the BitDepth dataset’s results. However, the average results for the Caicedo et al. dataset for both image bit depths in comparison to the average results for the BitDepth datasets were superior with large margins for all three evaluation indexes. As the same segmentation model was used for both dataset, it can be inferred that in general, images in the Caicedo et al. were easier to segment in comparison to the BitDepth dataset. Indeed, the Caicedo dataset contains images from a cell culture, where nuclei are usually relatively homogeneous in terms of shape or size, while our dataset was based on tissue sections, where nuclei of different cell types were present.

With a more in-depth analysis of the Caicedo et al. segmentation results, we noted three cases with zero Dice, AJI and PQ scores. For these three images, there were no nuclei in the images and ground truth segmentation masks, but the model identified a few nuclei (as shown in Figure 7); thus, zero scores were achieved for these three images. The scores for these three images did not affect the average scores that much, but the SDs were changed by a large margin.

Our experimental results in Figure 8 show that with reducing the image bit depth to two or four bits, the nuclei instance segmentation performances degraded. However, from the image bit depth of six upward, similar average results can be observed. It should be noted that using or saving images with 2, 4 and 6 bits is not common practice, and we just reported the results to further investigate the impact of other image bit depth on the nuclei instance segmentation performance.

It is worth mentioning that using 8 bit images instead of 16 bit images did not change the training time or GPU usage in our experiments, as in both cases, 32 bit floating points were used in model training, which is the default training scheme in TensorFlow. Although it is possible to use mixed precision (using both 16 bit and 32 bit floating points) (https://www.tensorflow.org/guide/mixedprecision) (accessed on 26 May 2021) for model training, as we only used 8 bit and 16 bit images, again, this would not change the model performance, in terms of training time and GPU usage, in our conducted experiments.

All in all, the results from this study showed the very competitive nuclei segmentation performance of 8 bit and 16 bit images, which in practice may have two main applications: first, by reducing the storage space for saving WSIs (by saving them as 8 bit formats instead of 16 bit raw format) and, second, using 8 bit cameras instead of 16 bit cameras, which are faster for scanning. However, there are some limitations of this study that can be addressed in future works. Although we used two datasets in this work to report the results, the sizes of the datasets were rather small, which is a common problem for interpreting the DL-based results in medical image processing. Furthermore, we only explored the effect of image bit depth on a DL-based nuclei instance segmentation performance. However, in the case of using other image processing techniques (e.g., histogram-based image analysis or classical machine learning approaches), different outcomes may be observed. We explicitly investigate this impact on a DL-based approach, as supervised CNN models have been shown to deliver the best nuclei instance segmentation performances. Moreover, in this work, we used a single DL-based approach without using any sophisticated pre- and post-processing approaches to perform nuclei segmentation. Although our utilised segmentation model can deliver acceptable nuclei segmentation results (an extended version of our segmentation model achieved the first rank in the MoNuSAC 2020 post-challenge leaderboard for nuclei instance segmentation and classification (https://monusac-2020.grand-challenge.org/Results/) (accessed on 26 May 2021)), using other pre-processing steps such as DL-based foreground normalisation as suggested in [14], other segmentation models such as Hover-Net [32] or using test time augmentation and ensembling as post-processing [34] can lead to a slight improvement in the segmentation performance. However, delivering the best segmentation performance was not the main focus of this study. While we did not observe a significant impact of image bit depth on nuclei segmentation, it remains to be investigated whether the detection and segmentation of dynamic nuclei sub-compartments such as nucleoli, Cajal bodies and others [38] are influenced by image bit depth, which requires properly annotated datasets. Finally, it should be noted that in this study, we only investigated the impact of image bit depth on the nuclei segmentation performance, and any possible impact on other WSI analysis tasks such as gland detection or segmentation needs further investigation.

## 5. Conclusions

In this study, we investigated the impact of image bit depth on the performance of DL-based nuclei instance segmentation using different datasets. Our results showed very competitive nuclei instance segmentation performance for the models trained with 8 bit and 16 bit images, which suggested that processing 8 bit images seems sufficient for nuclei instance segmentation of the FS image. Exploring the impact of image bit depth on other WSI analysis tasks can be addressed in future studies.

## Figures and Tables

**Table 1:** Details of the BitDepth dataset.

Objective	Images Per Organ					#Total Images	#Nuclei	Avg #Nuclei Per Image
	#Bone	#Heart	#Kidney	#Liver	#Muscle			
20× air	1	2	2	2	2	9	1310	145.5
40× air	3	4	4	4	4	19	896	47.2
40× oil	4	4	4	4	4	20	762	38.1
63× oil	3	5	5	5	4	22	426	19.4
# sum	11	15	15	15	14	70	3394	48.5

**Table 2:** Fluorescence-stained nuclei instance segmentation performance of the BitDepth dataset based on image bit depth at different image magnifications. In cases, where the segmentation performance of 8 bit images differed significantly (*p*-values < 0.05) from that of 16 bit images, the results are shown in bold.

Dataset	Dice (%)		AJI (%)		PQ (%)	
	8 bit	16 bit	8 bit	16 bit	8 bit	16 bit
Data 1 (20× air)	87.4 ± 4.3	87.8 ± 3.4	66.4 ± 11.6	67.4 ± 11.5	62.9 ± 10.7	62.30 ± 10.8
Data 2 (20× air)	87.5 ± 4.1	88.1 ± 3.0	67.1 ± 10.8	67.1 ± 10.3	62.7 ± 11.0	62.4 ± 10.2
Data 3 (20× air)	87.5 ± 3.6	87.0 ± 2.9	67.4 ± 10.9	67.6 ± 10.4	62.3 ± 10.1	62.0 ± 10.5
Data 4 (20× air)	86.8 ± 4.6	87.7 ± 4.1	68.1 ± 11.6	68.9 ± 10.3	62.5 ± 12.6	64.8 ± 9.8
Data 1 (40× air)	88.1 ± 4.9	87.8 ± 5.0	65.5 ± 12.7	65.3 ± 12.8	61.6 ± 13.8	62.9 ± 12.2
Data 2 (40× air)	88.3 ± 4.2	88.1 ± 4.1	64.0 ± 15.0	65.4 ± 12.3	61.3 ± 14.7	61.0 ± 12.0
Data 3 (40× air)	88.4 ± 4.3	88.0 ± 4.5	66.4 ± 12.5	66.6 ± 11.6	62.6 ± 12.8	62.6 ± 11.3
Data 4 (40× air)	**88.1 ± 4.2**	**88.4 ± 4.1**	**66.7 ± 11.9**	**68.2 ± 11.8**	61.0 ± 12.0	62.8 ± 11.9
Data 1 (40× oil)	87.9 ± 6.0	88.7 ± 5.4	**62.4 ± 12.9**	**68.9 ± 13.6**	57.4 ± 14.4	62.7 ± 15.4
Data 2 (40× oil)	88.4 ± 5.8	88.4 ± 6.2	**61.7 ± 10.7**	**66.4 ± 12.4**	**55.6 ± 12.9**	**61.9 ± 12.3**
Data 3 (40× oil)	87.4 ± 5.9	87.1 ± 7.4	67.3 ± 11.9	67.8 ± 13.4	61.1 ± 11.3	61.1 ± 13.4
Data 4 (40× oil)	88.3 ± 5.6	88.0 ± 5.2	70.7 ± 12.7	67.1 ± 12.7	**64.8 ± 14.1**	**60.4 ± 13.1**
Data 1 (63× oil)	86.7 ± 8.1	85.8 ± 9.9	65.0 ± 15.2	64.2 ± 16.5	56.3 ± 15.2	55.1 ± 14.8
Data 2 (63× oil)	86.2 ± 5.5	86.7 ± 7.0	63.6 ± 13.2	64.0 ± 14.7	51.4 ± 13.5	56.0 ± 14.9
Data 3 (63× oil)	88.5 ± 4.6	88.9 ± 4.2	67.4 ± 13.7	64.6 ± 11.4	58.3 ± 13.9	54.6 ± 12.4
Data 4 (63× oil)	89.4 ± 3.9	89.6 ± 4.8	68.5 ± 13.5	69.4 ± 13.2	59.2 ± 14.7	61.6 ± 13.2

**Table 3:** Derived *p*-values from comparing 8 bit and 16 bit images of the BitDepth dataset. *p*-values < 0.05 are shown in bold in accordance with the results of Table 2.

Dataset	*p*-Values Dice	*p*-Values AJI	*p*-Values PQ
Data 1 (20× air)	0.82	0.57	0.82
Data 2 (20× air)	0.73	0.99	0.99
Data 3 (20× air)	0.13	0.99	0.50
Data 4 (20× air)	0.06	0.36	0.13
Data 1 (40× air)	0.44	0.87	0.06
Data 2 (40× air)	0.78	0.27	0.87
Data 2 (40× air)	0.35	0.81	0.99
Data 2 (40× air)	**0.03**	**0.02**	0.06
Data 1 (40× oil)	0.10	**0.03**	0.23
Data 2 (40× oil)	0.88	**0.01**	**0.004**
Data 3 (40× oil)	0.97	0.99	0.88
Data 4 (40× oil)	0.39	0.06	**0.03**
Data 1 (63× oil)	0.85	097	0.57
Data 2 (63× oil)	0.41	0.70	0.07
Data 3 (63× oil)	0.29	0.15	0.22
Data 4 (63× oil)	0.55	0.37	0.11

## Data Availability

Two datasets were used in this study. One is the proposed dataset, which is available at https://github.com/masih4/BitDepth_NucSeg (accessed on 26 May 2021). We also used another publicly available dataset [10], which is available at https://bbbc.broadinstitute.org/BBBC039 (accessed on 26 May 2021).

## Abbreviations

DAPI	4${}^{\prime }$,6-Diamidino-2-phenylindole
DL	Deep learning
FS Image	Fluorescence-stained image
H&E staining	Hematoxylin and eosin staining
IHC staining	Immunohistochemical staining
CNN	Convolutional neural network
WSI	Whole-slide image
FOV	Field of view
AJI	Aggregate Jaccard index
PQ	Panoptic quality
TP	True positive
FN	False negative
FP	False positive
SD	Standard deviation
